# Colon cancer survival prediction from gland shapes within histology slides using deep learning

**DOI:** 10.1515/jib-2024-0052

**Published:** 2025-07-14

**Authors:** Rawan Gedeon, Atulya Nagar

**Affiliations:** Faculty of Applied Science, Technology and Engineering, Technology Department, 61180 Bethlehem University, Bethlehem, Palestine; School of Mathematics, Computer Science and Engineering, Liverpool Hope University, Hope Park, Liverpool, L16 9JD, UK

**Keywords:** deep learning, gland segmentation, colorectal cancer, survival analysis, TCGA, morphological features

## Abstract

This study investigates the application of deep learning techniques for segmenting glands in histopathological images of colorectal cancer. We trained two convolutional neural network models, U-Net and DCAN, on a combination of the GlaS and CRAG datasets to enhance generalization across diverse histological appearances, selecting DCAN for its superior accuracy in delineating gland boundaries. The goal was to achieve robust gland segmentation applicable to whole slide images (WSIs) from The Cancer Genome Atlas (TCGA). Using the segmented glands, we extracted patient-level morphological features and used them to predict survival outcomes. A Cox proportional hazards model was trained on these features and achieved a high concordance index, indicating strong predictive performance. Patients were then stratified into high- and low-risk groups, with significant differences in survival distributions (log-rank *p*-value: 0.01317). In addition, we benchmarked our models against state-of-the-art gland segmentation methods on GlaS and CRAG, highlighting the trade-off between domain-specific accuracy and cross-dataset robustness.

## 1 Introduction

Colorectal cancer (CRC) is a malignant epithelial tumour originating in the large bowel. CRC is graded as well-, moderately-, and poorly-differentiated on the basis of the percentage of gland formation. Well-differentiated tumours are mostly glandular, while poorly-differentiated tumours glands are seriously deformed. Well- and moderately- differentiated carcinomas are referred to as low grade, and poorly-differentiated as high grade. High grade is linked to a low probability of survival [1].

The Cancer Genome Atlas (TCGA) project is a cancer genomics program that includes molecular profiling for a range of cancer types. In addition to the molecular and clinical data, TCGA contains Whole Slide Images (WSIs) of the resection specimens from CRC patients with primary carcinoma. Pathologic examination of resection specimens is crucial to plan patient treatment; pathologists also investigate gland formation to decide on the histologic tumour grading [2]. In this research, we process a subset of colorectal adenocarcinoma (COAD) patient cohort in order to investigate how gland morphology within WSIs can predict the patient risk.

## 2 Related works

Survival studies take different approaches in histopathology. Some predict the patient risk based on a region of interest (ROI) [3], while others predict the risk based on the entire WSI [4]. Some studies use deep learning models to process images and then extract morphological features of the processed objects [5], while others use deep learning models to directly predict the risk of the patient [3]. The goal of employing deep learning into survival studies is to investigate their capability to discover new prognosticators that are significant to patients’ outcomes. Kather et al. [3] identified different tissue types in a ROI in WSIs using a deep convolutional neural network (CNN) and aggregated each of these tissue types into a prognostic score to predict CRC patients’ overall survival (OS). Zhu et al. [4] argue that a region cannot fully represent the patients’ survival status due to the heterogeneity of the tumour. They propose a Whole Slide Histopathological Images Survival Analysis framework (WSISA) that samples hundreds of candidate patches randomly from WSIs and distinguishes survival-discriminative patterns by grouping them into different clusters. They train a deep convolutional survival model (DeepConvSurv) on each cluster, calculate patient features from selected clusters and then train an aggregation model to make a final survival prediction. They conducted experiments on glioma and non-small-cell lung cancer, and their results showed that their methodology improved the prediction performance compared to other methods. Bychkov et al. [6] directly predicted five-year disease-specific survival (DSS) for CRC patients from the tumour tissue microarray (TMA) samples without classifying the tissues. A set of features for each tile in the TMA was extracted using a VGG-16 network [7] pre-trained on ImageNet [8] and then inputted to a Long Short-Term Memory (LSTM) [9] to predict a patient probability of five-year survival. Wulczyn et al. [10] presented a comprehensive study that predicts survival across ten cancer types from TCGA. Their deep learning system consisted of multiple CNNs that extract features from uniformly sampled patches from the patient WSIs. Features were then averaged, and fed to a fully connected layer to output a probability over discretized survival times. Their study demonstrated that deep learning can predict survival directly from histology slides without any expert annotation; however, a large dataset is needed.

Prior works have used either annotated regions of interest or randomly sampled regions to predict patient survival. This paper builds upon our earlier research, presented at PACBB, where we introduced a workflow for predicting patient survival based on epithelial nuclei morphological features extracted from a single region in the WSI. In this extended version, we enhance the approach by predicting overall survival using gland shapes from the entire WSI, allowing us to capture the heterogeneity of gland structures. We predict a risk score based on the morphological features of these glands. Gland segmentation is performed using a deep contour-aware network (DCAN) [11], which provides clear contours for separating clustered glands.

Instance gland segmentation is a challenging task due to many reasons. First, large differences of glandular shape exist among benign and malignant cases; glands become seriously deformed as the grade of cancer progresses. Second, separating glands can be difficult as they can be touching each other. U-Net [12] achieved excellent performance on the gland segmentation task. To get more accurate segmentation, Chen et al. [11] proposed DCAN. DCAN formulates the gland segmentation task as a multitask learning problem where the gland objects and contours probability maps are upsampled with two different branches simultaneously. Each branch integrates multi-level contextual features in order to produce good probability maps. To produce the final segmentation maps, both probability maps are fused together. Xu et al. [13] proposed a framework for gland instance segmentation to fuse complex multichannel regional and boundary patterns with side supervision. Xu et al. [14] enhanced this framework to include additional bounding box information. Raza et al. [15] presented a Multi-Input-Multi-Output network (MIMO-Net) for gland segmentation and achieved state-of-the-art performance.

## 3 Workflow

**Figure 1:**
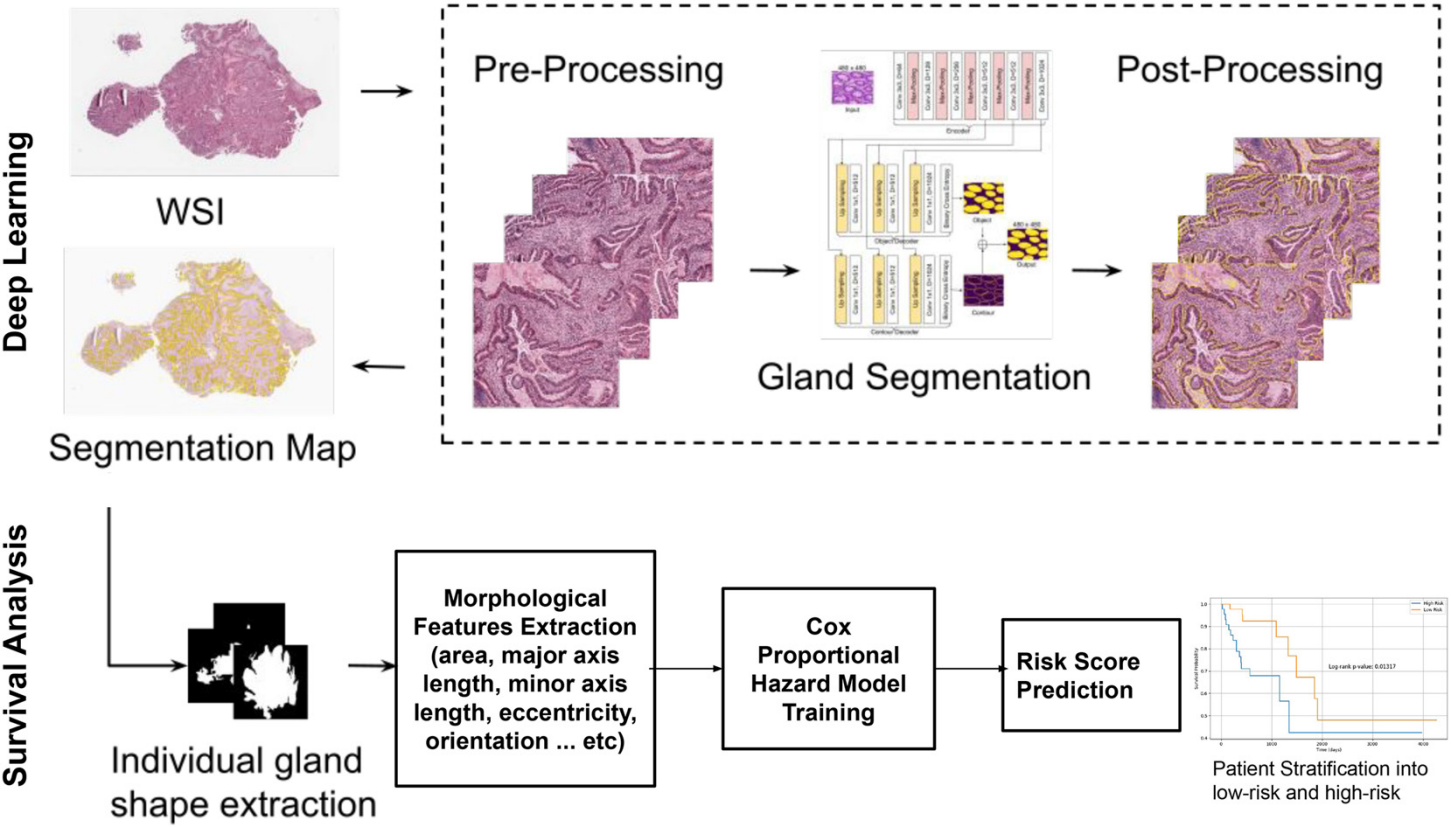
The study workflow.

The workflow of our study is split into two main parts and is demonstrated in Figure 1. First, glands within WSIs are segmented using DCAN, then we do survival prediction based on glands morphological features.

### 3.1 Instance gland segmentation

We trained and evaluated two convolutional neural network models for gland segmentation: U-Net [12] and DCAN [11]. While both models are widely used for biomedical image segmentation, we selected DCAN for subsequent feature extraction and survival prediction, as it outperformed U-Net across in terms of segmentation accuracy and boundary delineation.

DCAN effectively captures multi-level contextual features, leveraging different receptive field sizes to handle the variability in gland morphology, particularly in malignant cases where glands exhibit irregular shapes. The network architecture consists of a downsampling path for feature extraction and an upsampling path for pixel-wise mask prediction. It integrates multi-scale feature maps through bilinear upsampling, enabling precise segmentation across varying gland shapes and sizes.

DCAN’s multi-task learning approach enhances its generalisation ability by simultaneously learning gland object segmentation and contour detection. This helps to separate touching glands, especially in benign cases. The model is trained using cross-entropy loss, with deep supervision applied to guide the training process. The total loss function includes both gland object and contour segmentation losses, with auxiliary losses discounted as training progresses.

Following segmentation, small regions (less than 400 pixels) are removed, and smoothing and hole-filling post-processing steps are applied to refine the final mask.

#### 3.1.1 Datasets

To develop a segmentation model capable of generalizing to unseen WSIs from real-world clinical sources such as TCGA, we adopted a cross-dataset training strategy using two public histopathology datasets: the Gland Segmentation (GlaS) challenge dataset [16] and Colorectal Adenocarcinoma Gland (CRAG) dataset [17], 18]. GlaS was introduced in the gland segmentation MICCAI challenge in 2015, scanned at 20× magnification with a pixel resolution of 0.465 μm/pixel. GlaS is comprised of 165 images, split into 85 training (37 benign and 48 malignant) and 80 test images (37 benign and 43 malignant) split into Test A and Test B. Most images are of size 775 × 522 pixels and all images have associated instance-level segmentation ground truth that precisely delineates the gland contours. CRAG has a total of 213 H&E CRA images taken from 38 whole slide images (WSIs) scanned with a pixel resolution of 0.55 μm/pixel equivalent to 20× magnification. The 38 WSIs belong to different patients and are mostly of size 1,512 × 1,516 pixels, with corresponding instance-level ground truth. The CRAG dataset is split into 173 training images and 40 test images with different cancer grades.

#### 3.1.2 Implementation details

The framework was implemented using Pytorch. Weights were initialised using Xavier initialisation with a uniform distribution. We stain normalised the images using Vahadene stain normalisation method. We then extracted overlapping patches of size 500 × 500, a total of 6,642 formed our training set. During training, we randomly rotated the image using (0°, 90°, 180°, 270°), flipped the image horizontally with 0.5 probability, and cropped a random image patch of size 480 × 480 pixels. We used stochastic gradient descent with an initial learning rate of 10^−4^ and a batch size of 4. We trained the model for 78 epochs.

#### 3.1.3 Evaluation

To evaluate performance, we tested the model on each dataset’s test split individually, as well as on the combined test set. We evaluated the performance of our model using the same evaluation criteria of the MICCAI GlaS challenge that consists of *F*_1_ score, object-level dice and object-level Hausdorff distance. The *F*_1_ score measures the detection accuracy of each gland object. The dice index measures segmentation quality between the predicted segmentation output and the ground truth. The Hausdorff distance measures the shape similarity between the shape of the segmented object and ground truth object.

## 4 Application

### 4.1 Patient cohort

Diagnostic WSIs for colon cancer patients were retrieved for the TCGA COAD cohort with their clinical data from the Genomic Data Commons portal. We processed 161 patients of this patient cohort, each patient had one WSI. A clinical information summary is provided in Table 1. Survival time is available from the date of the diagnosis. The median patient age for patients is 70 years. Approximately 52 % of the patients are male, 50 % are at stage I/II, and the remaining are at stage III/IV.

**Table 1:** Summary of clinical parameters.

Clinical parameters		Full cohort
No. of patients		161
Age (years)		70
Overall survival (months)		20.67
Gender	Male	84
	Female	77
Tumour stage	Stage I	21
	Stage II	59
	Stage III	44
	Stage IV	29
Patient status	Alive	86
	Dead	75

### 4.2 WSI processing

We segmented the glands in a WSI by extracting non-overlapping patches in a sliding window approach from the tissue region. The background area was not processed. Since the gland segmentation model was trained with images at 20× objective magnification, we extracted patches to match the same magnification level. If level zero magnification is 40×, we down-sampled the patch by a factor of 2. We grouped a few patches to perform inference in a batch. Patches were also stain normalised using the same target image used during training. Post-processing operations followed where a smoothing operation with a disk filter of radius 3 and filling in holes in the segmentation were performed.

### 4.3 Extracting morphological features from segmented glands

From each segmented gland, we extracted morphological features, including area, major axis length, minor axis length, eccentricity, orientation, convex area, filled area, equivalent diameter, solidity, extent, perimeter, box aspect ratio, and compactness. These features capture the essential geometric properties of the glands, which have been shown to correlate with tumour behaviour.

Since each patient had multiple glands in the WSI. The extracted morphological features were aggregated at the patient level to generate a feature vector. Aggregation methods included calculating the mean, median, standard deviation, skewness, kurtosis, and maximum of each feature across all glands for a given patient.

### 4.4 Survival analysis

The Cox Proportional Hazards Model was employed to assess the relationship between gland morphological features and survival outcomes. To ensure the robustness of the model, we implemented 5-fold cross-validation to split the data into training and test sets. A Cox Proportional Hazards model was fitted on the training data to estimate the relationship between the features and the survival times. We used regularisation to prevent the model from over-fitting. For each fold, we predicted risk scores for the test set and we calculated the C-index, a performance metric indicating how well the model predicts survival time.

Patients were then grouped into “High Risk” or “Low Risk” based on the median risk score. Kaplan–Meier survival curves were generated for each stratum to visually assess survival differences. Log-rank tests were performed to determine the statistical significance of these groups.

### 4.5 Gland segmentation performance

Table 2 presents the performance of U-Net and DCAN models on gland segmentation across multiple test datasets, using the F1 score, object-level Dice, and object-level Hausdorff distance as evaluation metrics. On GlaS Test A, both models achieved relatively high scores, with DCAN slightly outperforming U-Net in terms of F1 score (0.785 vs. 0.766) and yielding a lower Hausdorff distance (73.13 vs. 76.51), suggesting better boundary localization. For GlaS Test B, which is known to be more challenging due to increased staining and structural variability, both models achieved the same F1 score (0.723), but U-Net obtained a slightly higher Dice score, while DCAN showed marginally better boundary precision with a lower Hausdorff distance. When combining GlaS Test A and B, DCAN consistently outperformed U-Net in all three metrics, confirming its robustness across varying sample complexities. DCAN outperformed U-Net on CRAG with a higher F1 score (0.674 vs. 0.618), better Dice index, and significantly lower Hausdorff distance (149.51 vs. 173.97), indicating stronger generalization.

**Table 2:** Performance comparison of U-Net and DCAN on gland segmentation.

Dataset	Model	F1 score ↑	Object-level Dice ↑	Object-level Hausdorff ↓
GlaS Test A	U-Net	0.766	0.798	76.512
	DCAN	0.785	0.799	73.134
GlaS Test B	U-Net	0.723	0.796	56.861
	DCAN	0.723	0.789	56.716
GlaS A + B (combined)	U-Net	0.755	0.797	71.599
	DCAN	0.769	0.796	69.029
CRAG test set	U-Net	0.618	0.741	173.973
	DCAN	0.674	0.755	149.514
All test sets combined	U-Net	0.710	0.778	105.724
	DCAN	**0.737**	**0.783**	**95.857**

F1 score: harmonic mean of precision and recall; balances false positives and false negatives. Object-level Dice: measures overlap between predicted and true segmented objects, accounting for correct object matching. Object-level Hausdorff distance: quantifies the boundary-based dissimilarity between individual segmented objects and their corresponding ground truth objects.

When aggregating all test sets, DCAN maintained its superiority, achieving higher overall F1 (0.737 vs. 0.710) and Dice (0.783 vs. 0.778), along with a lower average Hausdorff distance. These results suggest that DCAN is more effective than U-Net in both segmentation accuracy and shape consistency, particularly when tested across diverse datasets.

**Figure 2:**
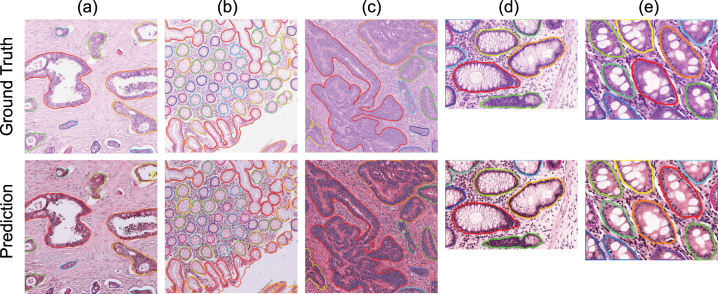
Visual gland segmentation results on CRAG(a, b, c) and GlaS(d, e) test dataset.

To complement the quantitative evaluation, Figure 2 presents qualitative results from DCAN on representative test images – three from CRAG and two from GlaS. These examples visually demonstrate the model’s ability to delineate gland structures with high fidelity across datasets of varying complexity and staining characteristics.

**Figure 3:**
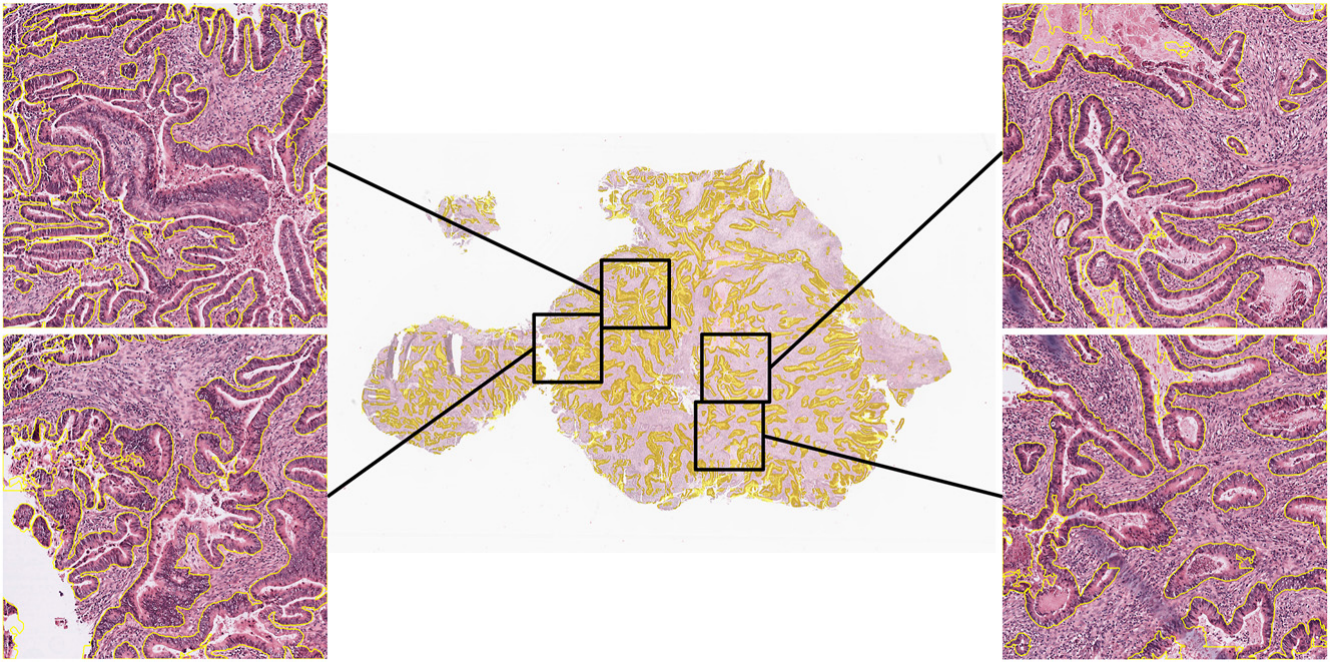
Visual gland segmentation result on a WSI from TCGA COAD patient cohort.

Figure 3 illustrates qualitative segmentation results produced by the DCAN model on a WSI from the TCGA-COAD patient cohort. Enlarged regions on the sides highlight detailed glandular structures along with the corresponding segmentation outputs. The visualization demonstrates DCAN’s ability to accurately delineate gland boundaries, even in complex tissue architecture, reinforcing its strong performance as observed in the quantitative evaluation.

#### 4.5.1 Benchmarking

To further contextualize the performance of our models, we benchmarked the results of our U-Net and DCAN models against several state-of-the-art methods: TA-Net [19], MicroNet [20], and DSE [21]. As shown in Table 3, our DCAN model achieved competitive results in terms of segmentation quality, particularly on the CRAG dataset, where it attained an object-level Dice of 75.5 and a Hausdorff distance of 149.5. While these results are lower than TA-Net and other recent architectures, they reflect strong generalization because our model was trained on a combined dataset (GlaS + CRAG) – a more heterogeneous and challenging training setup than dataset-specific training.

**Table 3:** Benchmark comparison on the GlaS and CRAG test sets. Existing models are trained and evaluated on individual datasets, while our U-Net and DCAN models are trained on the combined GlaS + CRAG training sets.

Method	GlaS (Test A + B)			CRAG		
	F1 ↑	Obj-Dice ↑	Obj-H ↓	F1 ↑	Obj-Dice ↑	Obj-H ↓
TA-Net [19]	90.5	90.2	50.8	84.2	89.3	105.2
DSE [20]	89.4	89.9	55.9	83.5	88.9	120.1
MicroNet [21]	86.5	87.6	70.4	82.5	87.5	160.1
DCAN (ours)	76.9	79.6	69.0	67.4	75.5	149.5

### 4.6 Survival analysis results

To assess the prognostic value of gland morphological features, we applied the Cox Proportional Hazards Model, using the aggregated patient-level features as covariates. We implemented 5-fold cross-validation to ensure the model’s robustness and prevent over-fitting. For each fold, the model was trained on 80 % of the data and tested on the remaining 20 %. The Concordance-index was used to evaluate the predictive accuracy of the model across each fold. The survival prediction model yielded a mean Concordance Index of 0.927 across all folds, demonstrating a strong correlation between the predicted risk scores and survival times. This suggests that the combination of morphological features was effective in predicting survival outcomes.

**Figure 4:**
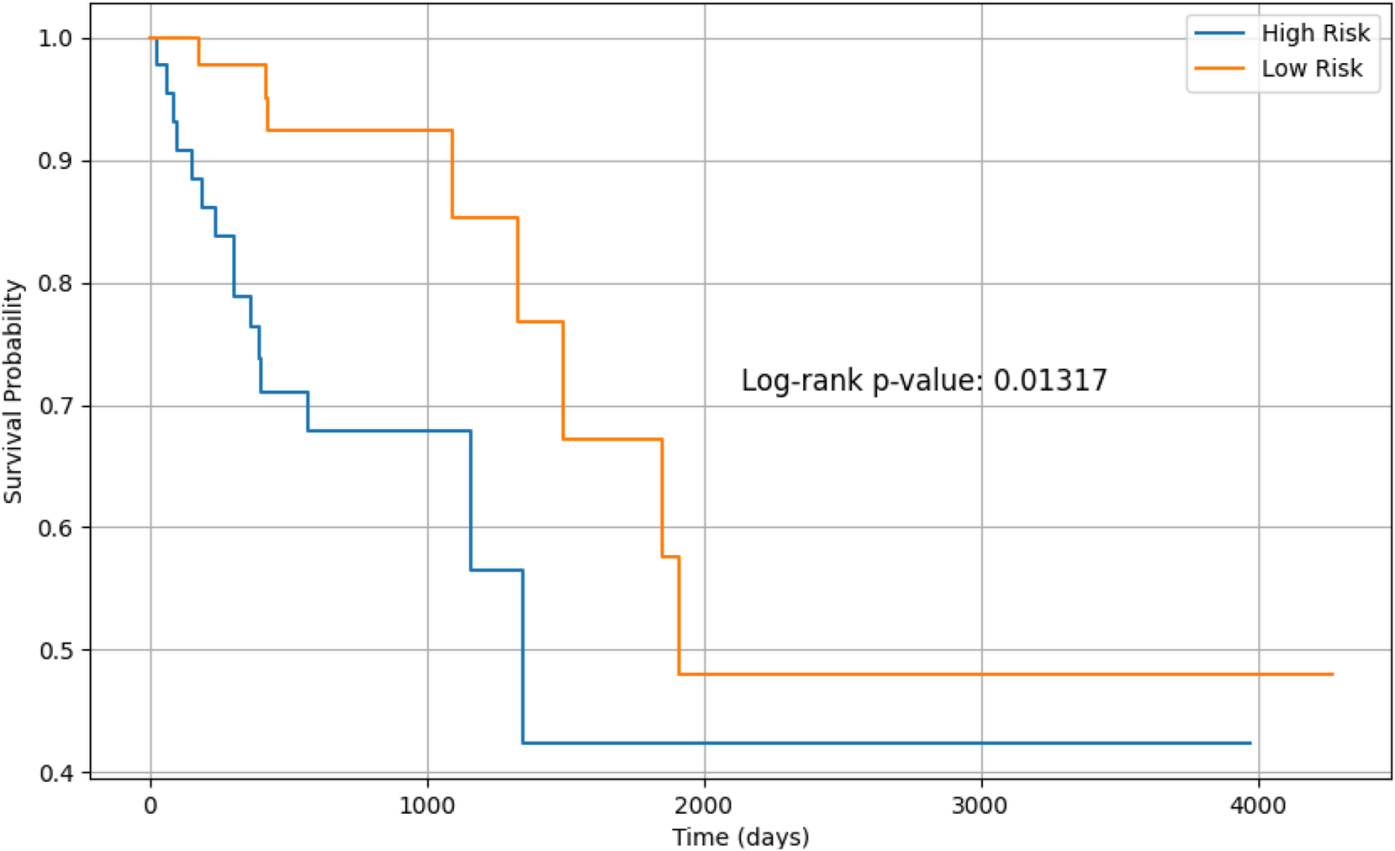
Kaplan–Meier survival curves by risk group.

To explore survival differences, patients were stratified into “High Risk” and “Low Risk” groups based on their predicted risk scores, using a median split. Kaplan–Meier survival curves for both groups, shown in Figure 4, illustrate distinct survival trajectories. The log-rank test confirmed that the differences were statistically significant (*p* = 0.01317).

## 5 Discussion

The findings from this study demonstrate the potential of combining deep learning-based gland segmentation with survival analysis to predict patient outcomes in colorectal cancer. Our model achieved a high Concordance Index (CI) of 0.927, indicating a strong agreement between the predicted risk scores and the actual survival times of the patients. This suggests that the morphological features extracted from segmented glands are robust predictors of survival.

The high CI emphasises the reliability of the model in distinguishing between patients with differing prognoses. A CI value closer to 1.0 reflects near-perfect concordance, showing that the model accurately assigns higher risk scores to patients with shorter survival times.

Furthermore, stratifying patients into low-risk and high-risk groups based on the predicted risk scores yielded a statistically significant *p*-value of 0.01317, confirming that the differences in survival between the two groups are unlikely to have occurred by chance. A *p*-value below 0.05 typically indicates strong evidence against the null hypothesis, validating the model’s ability to categorise patients into distinct survival risk categories. This significant stratification reinforces the clinical relevance of the model, as it can aid in identifying high-risk patients who may benefit from more aggressive treatments.

Beyond predictive performance, our results also highlight the practical advantages of training segmentation models on heterogeneous, multi-source datasets. By combining the GlaS and CRAG datasets during training, the model was exposed to a wide range of gland morphologies, and image resolutions. This diversity enhanced its robustness and enabled effective generalization to unseen WSIs from the TCGA-COAD cohort. Qualitative results on TCGA demonstrate that the model is capable of accurately delineating glands in real-world clinical samples, despite inter-domain variability. These findings underscore the importance of multi-domain training for developing segmentation systems that are deployable in diverse clinical settings.

Taken together, these results emphasize the value of integrating morphological features into survival prediction models and support their potential contribution to personalized treatment planning. However, there are several limitations to our approach. The TCGA dataset, while large and well-curated, may not fully represent the diversity of colorectal cancer patients, particularly in terms of demographic factors such as ethnicity or socioeconomic status.

Future work will aim to validate the survival results on additional independent cohorts to further assess their generalizability. We also plan to explore the integration of complementary data modalities, such as genomic or molecular biomarkers, into the predictive framework.

In conclusion, this study demonstrates the feasibility and effectiveness of using deep learning models for gland segmentation in histopathological images combined with survival analysis to predict colorectal cancer patient outcomes. The high Concordance Index and significant *p*-value underscore the model’s potential as a valuable tool in clinical decision-making.
